# The cell envelope subtilisin-like proteinase is a virulence determinant for *Streptococcus suis*

**DOI:** 10.1186/1471-2180-10-42

**Published:** 2010-02-10

**Authors:** Laetitia Bonifait, Maria de la Cruz Dominguez-Punaro, Katy Vaillancourt, Christian Bart, Josh Slater, Michel Frenette, Marcelo Gottschalk, Daniel Grenier

**Affiliations:** 1 Groupe de Recherche en Écologie Buccale, Faculté de Médecine Dentaire, Université Laval, Quebec City, Quebec, Canada; 2 Groupe de Recherche sur les Maladies Infectieuses du Porc, Faculté de Médecine Vétérinaire, Université de Montréal, Saint-Hyacinthe, Quebec, Canada; 3 The Royal Veterinary College, Hatfield, UK

## Abstract

**Background:**

*Streptococcus suis *is a major swine pathogen and zoonotic agent that mainly causes septicemia, meningitis, and endocarditis. It has recently been suggested that proteinases produced by *S. suis *(serotype 2) are potential virulence determinants. In the present study, we screened a *S. suis *mutant library created by the insertion of Tn*917 *transposon in order to isolate a mutant deficient in a cell surface proteinase. We characterized the gene and assessed the proteinase for its potential as a virulence factor.

**Results:**

Two mutants (G6G and M3G) possessing a single Tn*917 *insertion were isolated. The affected gene coded for a protein (SSU0757) that shared a high degree of identity with *Streptococccus thermophilus *PrtS (95.9%) and, to a lesser extent, with *Streptococcus agalactiae *CspA (49.5%), which are cell surface serine proteinases. The SSU0757 protein had a calculated molecular mass of 169.6 kDa and contained the catalytic triad characteristic of subtilisin family proteinases: motif I (Asp_200_), motif II (His_239_), and motif III (Ser_568_). SSU0757 also had the Gram-positive cell wall anchoring motif (Leu-Pro-X-Thr-Gly) at the carboxy-terminus, which was followed by a hydrophobic domain. All the *S. suis *isolates tested, which belonged to different serotypes, possessed the gene encoding the SSU0757 protein. The two mutants devoid of subtilisin-like proteinase activity had longer generation times and were more susceptible to killing by whole blood than the wild-type parent strain P1/7. The virulence of the G6G and M3G mutants was compared to the wild-type strain in the CD1 mouse model. Significant differences in mortality rates were noted between the P1/7 group and the M3G and G6G groups (*p *< 0.001).

**Conclusion:**

In summary, we identified a gene coding for a cell surface subtilisin-like serine proteinase that is widely distributed in *S. suis*. Evidences were brought for the involvement of this proteinase in *S. suis *virulence.

## Background

The swine pathogen *Streptococcus suis *is transmitted via the respiratory route and colonizes the palatine tonsils and nasal cavities of pigs from where it can disseminate throughout the animal and cause infections [1], mainly septicemia, meningitis, and endocarditis, as well as arthritis [1]. Zoonotic infections occur mainly in individuals who work in close contact with pigs or pork by-products [2]. In fact, *S. suis *is considered one of the most important etiologic agents of adult meningitis in Asian countries [3]. While thirty-five serotypes (1 to 34 and 1/2) have been identified based on capsular antigens, serotype 2 is considered the most virulent and is the most commonly recovered from diseased pigs and humans [1]. Over the past ten years, numerous studies have been undertaken to identify putative virulence factors in *S. suis *[1,4,5]. Among these virulence factors, the polysaccharide capsule, which provides protection against phagocytosis [6], appears to be essential for the pathogenicity of *S. suis*. However, considering the multi-step pathogenesis of *S. suis *infections, it is likely that the virulence of this pathogen is determined by more than one factor [7].

Proteases, which are hydrolytic enzymes that catalyze the cleavage of peptide bonds, are critical virulence factors for numerous microbial pathogens [8]. These enzymes hydrolyze a variety of host proteins, including serum and tissue components, thus helping to neutralize the host immune defense system and causing tissue destruction and invasion [8]. Interestingly, these enzymes show great potential as vaccine antigens and are promising targets for the development of anti-bacterial drugs [9]. A previous study in our laboratory identified four proteolytic enzymes produced by *S. suis*, including one on the cell surface that degrades a chromogenic substrate highly specific for chymotrypsin-like proteases [10]. In the present study, we screened an *S. suis *P1/7 (serotype 2) mutant library created by the insertion of Tn*917 *transposon in order to isolate a mutant deficient in this activity. We characterized the gene and assessed the proteinase for its potential as a virulence factor.

## Methods

### Bacteria and mutant library

*S. suis *P1/7, a virulent serotype 2 European reference strain isolated from a pig with meningitis for which the genome has been sequenced by the *S. suis *Sequencing Group at the Sanger Institute [11], was used as the wild-type strain. Bacteria were routinely grown in Todd Hewitt broth (THB; BBL Microbiology Systems, Cockeysville, MA, USA) at 37°C under aerobiosis. A mutant library was constructed in a previous study [12] using the pTV408 temperature-sensitive suicide vector to deliver the Tn*917 *transposon into *S. suis *P1/7 via electroporation. To maintain the selective pressure during the growth of the mutants, the culture medium was supplemented with 1 μg/ml of erythromycin. *Escherichia coli *MC1061 (*hsdR2 hsdM+ hsdS+ araD139 Δ(ara-leu)7697 Δ(lac)X74 galE15 galK16 rpsL (StrR) mcrA mcrB1*), which was used for plasmid rescue, was grown in LB medium containing 100 μg/ml of erythromycin.

### Isolation of mutants deficient in proteinase activity

Mutants from the Tn*917 *library were individually grown overnight in THB and suspended in phosphate-buffered saline (PBS, 50 mM, pH 7.2) to an optical density of 1.0 at 660 nm (OD_660_). Bacterial suspensions (100 μl) were added to the wells of 96-well microplates along with 20 μl of the chromogenic substrate N-succinyl-Ala-Ala-Pro-Phe-*p*Na (2 mg/ml in 50% dimethyl formamide) (Sigma-Aldrich Canada Ltd., Oakville, ON, CANADA). This substrate is highly specific for subtilisin-like [13] and chymotrypsin-like enzymes [14]. The reaction mixtures were incubated at 37°C for 4 h. The release of *p*NA was quantified by measuring the absorbance at 415 nm (A_415_).

### Demonstration of transposon insertion and stability of mutants

Chromosomal DNA was isolated from cells harvested from overnight bacterial cultures as previously reported [15], except that proteinase K (Sigma-Aldrich Canada Ltd.) was used instead of protease I. The DNA was digested with *Hind*III restriction endonuclease, Southern blotted, and hybridized using a digoxigenin (DIG)-labeled probe specific for the *erm *gene in the Tn*917 *transposon as previously reported [12]. Hybridization was performed at 68°C, and the probe was detected using the NBT (*p*-nitroblue tetrazolium chloride)/BCIP (5-bromo-4-chloro-3-indolyl phosphate) chromogen system. The probe was generated from pTRKL2T [16] by PCR using the ermF 5'-ACGAGTGAAAAAGTACTCAACC-3' and ermR 5'-ACCTCTGTTTGTTAGGGAATTG-3' primers and the DIG-PCR labeling mixture. The stability of the Tn*917*-induced mutation was investigated by performing overnight serial passages (up to 35) of the mutants in erythromycin-free THB prior to measuring the hydrolysis of the chromogenic substrate N-succinyl-Ala-Ala-Pro-Phe-*p*Na as described above.

### Plasmid rescue and sequencing of the insertion site

The site of the transposon insertions in the *S. suis *P1/7 genome was determined by plasmid rescue [12]. The genomic DNA of the selected mutants was isolated and digested using *Hind*III, ligated, and transformed into chemically competent *E. coli *MC1061. Transformants were selected on LB agar containing erythromycin. Plasmid DNA was then extracted from the *E. coli *cells and was sequenced using the Tn*917 *(5'-aGAGAGATGTCACCGTCAAGT-3') primer to determine the DNA sequence contiguous to Tn*917*.

### Characterization and comparative analysis of SSU0757

The theoretical pI and molecular mass of SSU0757 were determined using software available at http://www.scripps.edu/~cdputnam/protcalc.html. The alignment and comparison of the amino acid sequences of the subtilisin-like serine proteinases was performed using BioEdit software.

### Distribution of *SSU0757 *in *S. suis*

Selected *S. suis *strains were tested for the presence of the subtilisin-encoding gene (*SSU0757*): S428 (serotype 1), 31533 (serotype 2), 89-999 (serotype 2), S735 (serotype 2), 90-1330 (serotype 2), 65 (serotype 2), 89-4223 (serotype 2), 2651 (serotype 1/2), 4961 (serotype 3), Amy12C (serotype 5), 1078212 (untypeable), and 1079277 (untypeable). Except for strains 90-1330, 65 and 89-4223, which were isolated from healthy pigs, all other isolates were from diseased pigs. Cell lysates were prepared from bacterial colonies recovered from agar plates. The presence of the gene was determined by PCR using the SUB163 (5'-GTCAGCGAATCAGCCTCAGAAAGTCCCGTT-3') and SUB4436R (5'-CTTCATCTTTTTTGTCAGTGGCAGTATTTG-3') primers.

### Growth studies

The generation times of *S. suis *wild-type strain P1/7 and the proteinase-deficient mutants were determined by inoculating erythromycin-free THB with late-log phase cultures and monitoring growth at OD_660_. Generation times were calculated from the growth curves.

### Susceptibility to whole blood

Venous blood samples were collected from the antecubital vein of a human volunteer using the Vacutainer™ system and sterile endotoxin-free blood collection tubes containing 150 IU of sodium heparin (Becton Dickinson, Franklin Lakes, NJ, USA). Informed consent was obtained from the donor prior to the experiment. The protocol was approved by the Université Laval ethics committee. *S. suis *(wild-type parent strain and mutants) were cultivated to the early stationary growth phase at 37°C. The cells were harvested by centrifugation at 11,000 *g *for 10 min, suspended in RPMI-1640 medium to an OD_660 _of 0.1, and diluted 1:100 in RPMI-1640 medium. Whole blood (1 ml) was mixed with pig serum anti-*S. suis *(300 μl) and *S. suis *cells (100 μl). Anti-*S. suis *serum was prepared in pigs by injecting whole bacterial cells as previously described [17]. The mixtures were incubated for 4 h at 37°C with occasional gentle shaking. Infected whole blood cultures were harvested at 0 and 4 h. The first time point (0 h) was considered as the 100% viability control. Infected whole blood samples were 10-fold serially diluted (10^-1 ^to 10^-4^) in PBS and plated on Todd-Hewitt agar plates. After a 24-h incubation at 37°C, the number of colony forming units (cfu) was determined. The experiments were carried out in duplicate.

### Experimental infections in mice

Thirty-nine female six-week-old CD1 mice (Charles River Laboratories, Saint-Constant, QC, Canada) were acclimatized to a 12 h light/dark cycle and were given rodent chow and water ad libitum. On the day of the experiment, the mice (11 per group) were infected by i.p. injection of 1 ml of either *S. suis *wild-type strain P1/7 or the Tn*917 *mutants deficient in proteinase activity at a concentration of 7 × 10^7 ^CFU/ml in THB. Six control mice were inoculated with the vehicle solution (sterile THB) alone. The CD1 mouse has proven to be an excellent model of *S. suis *infections that result in an early septic shock-like syndrome and a second late phase *S. suis *infection that induces meningitis and brain damage [18-20]. The septicemic phase of *S. suis *infections is characterized by depression, rough hair coat, swollen eyes, weakness, and death during the first 48 h post-infection. If animals survive this critical step of the disease, they may still develop central nervous system damage and meningitis, with the sudden appearance of nervous signs beginning 3-4 days post-infection, including hyperexcitation, episthotonus, opisthotonus, bending of the head toward one side, and walking in circles [18]. Clinical signs of infection and survival were recorded on a daily basis post-infection for 14 days as previously described [18]. Mice exhibiting extreme lethargy or neurological signs were considered moribund and were humanely euthanized. All experiments involving mice were conducted in accordance with the guidelines and policies of the Canadian Council on Animal Care and the principles set out in the Guide for the Care and Use of Laboratory Animals, and were approved by the Animal Welfare Committee of Université de Montréal. Overall survival rates for the various groups were calculated using Kaplan-Meier plots. Survival curves were compared using the log-rank test with the Holm-Sidak method used to analyze multiple curves. A *p *< 0.05 was considered statistically significant. All analyses were performed using the Sigma Plot System (v.11; Systat Software, San Jose, CA, USA).

## Results

**Figure 1:**
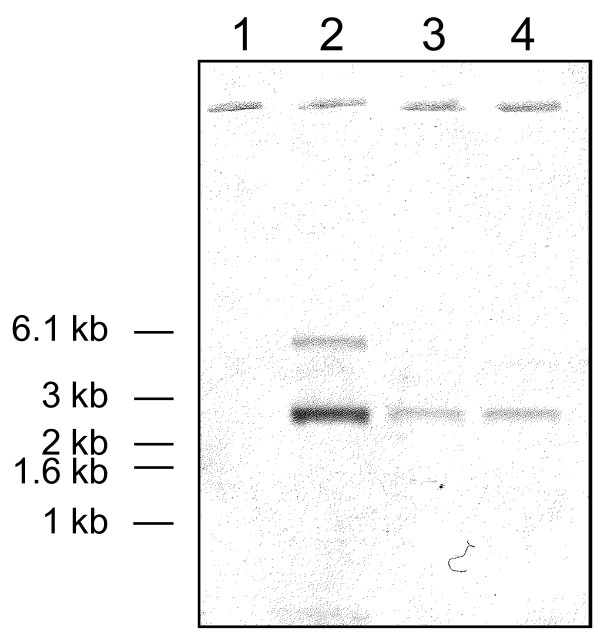
**Southern blot of *S. suis *P1/7 and the Tn*917 *mutants**. Chromosomal DNA was digested with *Hind*III restriction endonuclease and hybridized with a DIG-labeled probe specific for the *erm *gene. Lane 1, wild-type parent strain P1/7; lane 2, mutant J9G; lane 3, mutant M3G; lane 4, G6G.

The *S. suis *mutant library created by insertion of Tn*917 *transposon (1,200 mutants) was screened for degradation of the chromogenic substrate N-succinyl-Ala-Ala-Pro-Phe-*p*Na. Three mutants (G6G, J9G, and M3G) were found to be devoid of activity (A_415 _< 0.05) compared to parental strain (A_415 _= 0.85). With the objective to show that only one transposon insertion was present in mutants, chromosomal DNA was analyzed by Southern blotting using a DIG-labeled probe specific for the *erm *gene in the Tn*917 *transposon. As shown in Figure 1, only one Tn*917 *insertion occurred in the G6G and M3G mutants. Since the J9G mutant had two insertions, we only used the G6G and M3G mutants for further experiments. The mutations were highly stable, with G6G and M3G still unable to degrade the chromogenic substrate after 35 serial transfers in liquid medium (erythromycin-free).

**Figure 2:**
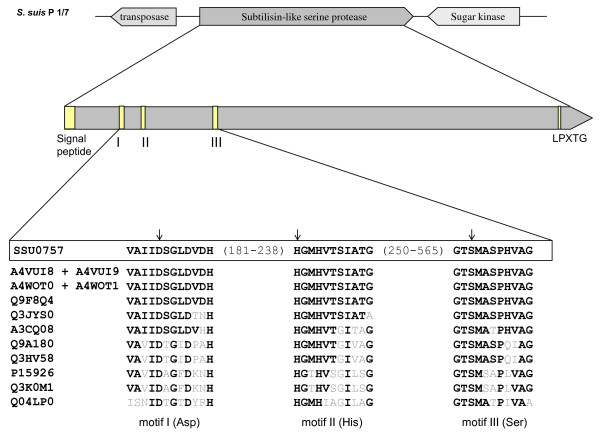
**Alignment of the catalytic triad (Asp_200 _- His_239 _- Ser_568_; indicated by arrows) of *S. suis *SSU057 and homologous streptococcal subtilisin-like proteinases**. Each catalytic triad is identified by UniProtKB accession numbers: A4VUI8 + A4VUI9 correspond to *S. suis *05ZYH33 (SSU05-0811 + SSU05-0812); A4WOT0 + A4WOT1 correspond to *S. suis *98HAH33 (SSU98-0811 + SSU98-0812); Q9F8Q4 corresponds to *S. thermophilus *PrtS; Q3JYS0 corresponds to *S. agalactiae *CspA; A3CQ08 corresponds to *S. sanguinis *PrtS; Q9A180 corresponds to *S. pyogenes *PrtS; Q3HV58 corresponds to *S. pyogenes *ScpC; P15926 corresponds to *S. pyogenes *ScpA; Q3K0M1 corresponds to *S. agalactiae *ScpB; Q04LP0 corresponds to *S. pneumoniae *PrtA.

**Figure 3:**
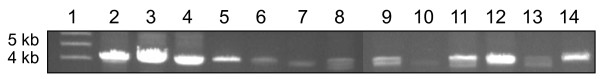
**Distribution of the gene coding for the SSU0757 protein in various *S. suis *strains**. Lane 1, DNA molecular weight markers; lane 2, S428 (serotype 1); lane 3, P1/7 (serotype 2); lane 4, 90-1330 (serotype 2); lane 5, S735 (serotype 2); lane 6, 65 (serotype 2); lane 7, 31533 (serotype 2); lane 8, 89-4223 (serotype 2); lane 9, 89-999 (serotype 2); lane 10, 2651 (serotype 1/2); lane 11, 4961 (serotype 3); lane 12, Amy12C (serotype 5); lane 13, 1078212 (untypeable); lane 14, 1079277 (untypeable).

To identify which gene was inactivated in mutants, the Tn*917 *insertion sites in G6G and M3G were sequenced. The affected gene corresponded to a gene coding for the SSU0757 protein in the genome of *S. suis *P1/7 based on a comparison of the sequence with those of the *S. suis *Sequencing Group at Sanger Institute. In mutant G6G, the transposon inserted at position 2,657 while in mutant M3G, it inserted at position 3,132. The analysis of the chromosomal region contiguous to the Tn*917*-inactivated gene confirmed that SSU0757 is not part of an operon. This and the transcriptional orientations of the contiguous genes suggested that there were no transposon-induced effects (Figure 2). This gene had a 4,758-nucleotide ORF and a G+C content of 41.64%, which was very similar to that of the *S. suis *genome (38-42%) [21]. There were also a transposase upstream and a sugar kinase downstream from the gene (Figure 2). To further explore the distribution of this gene in *S. suis*, we performed PCR assays using internal primers for the gene coding for SSU0757 using chromosomal DNA isolated from 11 strains belonging to serotypes 1, 1/2, 2, 3, and 5. Two untypeable isolates were also included. As shown in Figure 3, the gene was detected in all the strains tested, suggesting that it is widely distributed.

An *in silico *analysis of the *SSU0757 *gene product was performed to determine principal characteristics of the protein. This revealed that it corresponds to a 1,585-residue polypeptide with a predicted pI of 4.58 and a calculated molecular mass of 169.6 kDa. The protein contained the catalytic triad characteristic of subtilisin family proteinases: motif I (Asp_200_), motif II (His_239_), and motif III (Ser_568_). It also contained the Gram-positive cell wall anchoring motif (Leu-Pro-X-Thr-Gly) at the carboxy-terminus at positions 1551-1555 followed by a hydrophobic domain as well as an amino-terminal signal sequence with a putative cleavage site between residues 35 and 36 (Figure 2).

The alignment and comparison of the amino acid sequences of SSU0757 with known streptococcal subtilisin-like serine proteinases was performed using BioEdit software. The amino acid sequence of SSU0757 had a degree of identity of 98.9% and 98.4% with those of strains 05ZYH33 and 98HAH33, respectively. A database search revealed that the amino acid sequence of SSU0757 shared a high degree of identity (95.9%) with PrtS of *Streptococccus thermophilus*, which codes for a cell surface subtilisin-like proteinase (Table 1). As reported in Table 1, the SSU0757 protein shared significant identity with other streptococcal subtilisin-like proteinases. After the PrtS of *S. thermophilus*, the second highest degree of identity (49.5%) was with the CspA of *Streptococcus agalactiae*, which also codes for a cell surface subtilisin-like proteinase [22].

**Table 1:** Percentage identity of the amino acid (a.a.) sequences of *S. suis *P1/7 SSU0757 with proteinases from other streptococcal species.

Bacterial species	Accession no.	Predicted a.a. sequence % identity
*S. suis *uncharacterized protein	A4VUI8 + A4VUI9	98.9
*S. suis *uncharacterized protein	A4WOT0 + A4WOT1	98.4
*S. thermophilus *PrtS	Q9F8Q4	95.9
*S. agalactiae *CspA	Q3JYS0	49.5
*S. sanguinis *PrtS	A3CQ08	40.6
*S. pyogenes *PrtS	Q9A180	31.8
*S. pyogenes *ScpC	Q3HV58	31.8
*S. pyogenes *ScpA	P15926	24.0
*S. agalactiae *ScpB	Q3K0M1	23.6
*S. pneumoniae *PrtA	Q04LP0	16.2

**Figure 4:**
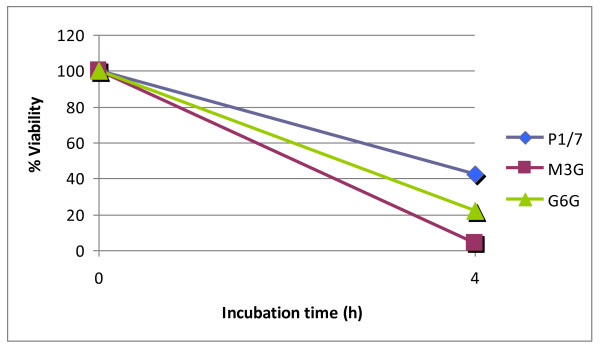
**Survival of *S. suis *wild-type strain P1/7 and mutants M3G and G6G in human whole blood**. Mixtures were incubated at 37°C for 4 h. A value of 100% was given to the colony forming units at time 0. Results are representative of two assays.

The role of the subtilisin-like proteinase of *S. suis *in nutrition was investigated by comparing the growth of the wild-type strain in THB with that of the Tn*917 *mutants. Table 2 lists the generation times for each strain. The two proteinase-deficient mutants had longer generation times than the wild-type strain. The impact of inactivating the proteinase on the survival of *S. suis *in human whole blood was also tested. As shown in Figure 4, the percent survival rate of the wild-type parent strain was 42.6 after a 4-h incubation in whole blood. The two mutants were much more sensitive, with a percent survival percent rate of 22.1 for G6G and 4.4 for M3G.

**Table 2:** Generation times of *S. suis *P1/7 and the Tn*917 *mutants deficient in the cell surface subtilisin-like proteinase.

Strain	Generation time in minutes (mean ± standard deviation)
P1/7	45.3 ± 6.9
M3G	57.6 ± 8.2
G6G	55.8 ± 4.8

**Figure 5:**
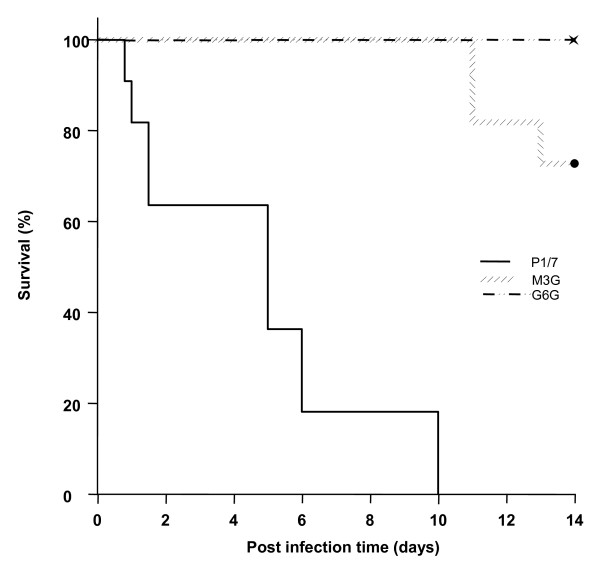
**Survival of mice inoculated with the wild-type strain P1/7, M3G, or G6G**. Six-week old CD1 mice were intraperitoneally inoculated with 7 × 10^7 ^cfu/ml and survival was recorded over a 14-day period. Data are expressed as the mean percentage of live animals in each group (n = 11).

The virulence of the G6G and M3G mutants was compared to the wild-type strain in the CD1 mouse model. All the animals in the P1/7 group presented severe clinical signs associated with septicemia and septic shock, including rough hair coat, depression, and prostration during the first 72 h post-infection. Four mice died from septicemia in this group (36.4%) (Table 3). From days 5-10, the rest of the mice infected with the P1/7 strain (63.6%) (Table 3) developed clear signs of neurological and/or vestibular symptoms, including hyperexcitation, episthotonus, opisthotonus, lateral bending of the head, walking in circles, rolling, and spinning [18]. Mice with these clinical signs were sacrificed for ethical reasons. M3G and G6G mice presented only mild clinical signs of a *S. suis *infection during the first 48 h post-infection, which mainly consisted of rough hair coat. Mice from both groups returned to their normal behavior after this period. Surprisingly, from days 11-13 post-infection, three mice from the M3G group (27.3%) died (Table 3). At this late stage of the trial, these deaths might have been due to either sub-clinical meningitis or endocarditis [18]. No deaths were recorded in the G6G group (Table 3). It is worth noting that *S. suis *was recovered from all the mice, whatever the group, that died either of septicemia or meningitis (data not shown). Survival curves for the various groups were analyzed using Kaplan-Meier plots and compared using the log-rank test with the Holm-Sidak method for analyzing multiple curves. Significant differences in mortality rates were noted between the P1/7 group and the M3G and G6G groups (*p *< 0.001) (Figure 5). In contrast, there were no statistical differences in mortality rates between the M3G and G6G groups (*p *> 0.05) (Figure 5).

**Table 3:** Virulence in CD1 mice of *S. suis *wild-type strain P1/7 and mutants M3G and G6G.

Strain	Death (%)*		Total mortality (%)
	Septicemia	Meningitis	
P1/7	36.4	63.6	100
M3G	0	27.3	27.3
G6G	0	0	0

* Eleven mice were infected per group and measurements were performed over a 14-day period post-infection. Percent of animals that died due to an infection or that were sacrificed for ethical reasons.

## Discussion

Bacterial pathogens possess various surface proteins, most of which are virulence determinants involved in attachment, multiplication, and invasion of the host. In the present study, we identified a *S. suis *gene that codes for a cell surface subtilisin-like proteinase containing the cell wall sorting signal LPXTG that is responsible for covalently anchoring proteins to cell wall peptidoglycan. The sortase A previously identified in *S. suis *has been reported to play an important role in anchoring LPXTG proteins to the cell wall [23] and may be involved in locating the subtilisin-like proteinase on the cell surface. A number of potential virulence factors previously characterized in *S. suis*, including the opacity factor [24], the virulence marker MRP [25], the surface antigen one [26], and a surface protein associated with invasion of porcine brain endothelial cells [20], contain the anchoring motif LPXTG,.

The cell surface subtilisin-like proteinase of S. suis showed the highest identity with the PrtS of S. thermophilus (95.9%) and the CspA of S. agalactiae (49.5%). In the dairy lactic bacterium S. thermophilus, the PrtS subtilisin-like proteinase degrades casein into peptides, which are required for efficient growth [27,28]. S. agalactiae is a major causal agent of mastitis in cattle [29] and is the principal cause of neonatal meningitis [30]. The CspA subtilisin-like proteinase of this pathogenic streptococcus is considered to be a critical virulence factor [22]. This proteinase has been shown to be involved in bacterial virulence in a neonatal rat sepsis model and in resistance to opsonophagocytic killing by human neutrophils in vitro [22]. More recently, the CspA of S. agalactiae has been shown to hydrolyze and inactivate CXC chemokines, many of which can recruit neutrophils to sites of infection [31].

Bacterial pathogenicity is a complex process that depends on the ability of the pathogen to multiply. The *S. suis *subtilisin-like proteinase appears to contribute to nutrient acquisition given that proteinase-deficient mutants had longer generation times than the parent strain *in vitro*. This is consistent with the study of Courtin et al. [28], who reported that the PrtS subtilisin-like proteinase of *S. thermophilus *is involved in nitrogen supply through casein hydrolysis. The mutants and the wild type strain were also compared for their ability to survive in human whole blood. We found that the parent strain was much more resistant to killing than the mutants. This suggests that the proteinase may degrade human serum proteins with bactericidal activity or opsonins involved in phagocytosis by immune cells. This is in agreement with the study of Harris et al. [22], who reported that the CspA subtilisin-like proteinase of *S. agalactiae*, which shares a high degree of identity with *S. suis*, contributes to the resistance to phagocytosis by neutrophils.

Given its cell surface localization, the subtilisin-like proteinase of S. suis may interact with host cells and induce an inflammatory response which is a feature of meningitis. Indeed, the S. suis proteinase may activate protease-activated receptors (PAR), which are members of the G protein-coupled receptors also known as seven-transmembrane domain receptors [32]. These receptors are found on several cell types and play an important role in inflammatory processes. More specifically, PAR-2 is known to be activated by serine proteases and bacterial proteinases [33]. Since S. suis cells are known to induce the production of pro-inflammatory cytokines by endothelial cells [34] and macrophages [35], part of this activation may be caused by the cell surface subtilisin-like proteinase identified in this study. Studies are currently in progress in our laboratory to verify this hypothesis. In a previous study, we reported that the presence of fibrinogen during growth of S. suis modulates its capacity to form a biofilm [36]. Given the ability of bacterial subtilisin-like proteinases to degrade fibrinogen [22,37,38], it may be hypothesized that the proteinase of S. suis could cleave fibrinogen leading to generation of fibrin, which may favor biofilm formation.

The contribution of the subtilisin-like proteinase to virulence was investigated in a mouse model. We found that the proteinase-deficient Tn917 mutants were significantly less virulent in mice. This clearly suggests that the S. suis subtilisin-like proteinase is an virulence determinant. Ge et al. [39] recently constructed a dipeptidyl peptidase IV deficient-mutant of S. suis and provided evidence for the critical role of this enzyme in the virulence of S. suis in a mouse model. This cell surface enzyme cleaves X-Pro/Ala dipeptides from the N-terminus of proteins but also possesses binding domains for fibronectin [39]. Given the involvement of the cell surface subtilisin-like serine proteinase in S. suis virulence, studies are in progress to clone this proteinase and determine whether it may represent a promising candidate for a protein-based vaccine.

## Conclusion

In summary, we identified a gene that codes for a cell surface subtilisin-like serine proteinase and that is widely distributed in *S. suis *strains. Evidences were brought for the involvement of this proteinase in *S. suis *virulence.

## Authors' contributions

KV and CB performed the molecular biology experiments. LB performed the growth study, determined the susceptibility to whole blood and helped to draft the manuscript. MCDP performed the animal study. JS constructed the Tn*917 *library. MG participated in the design of the study and helped to draft the manuscript. DG conceived the study and drafted the manuscript. All authors read and approved the final manuscript.
